# L'intérêt de la chirurgie micrographique dans la prise en charge du carcinome basocellulaire: expérience du service de dermatologie CHU Hassan II de Fès, Maroc

**DOI:** 10.11604/pamj.2019.33.245.18562

**Published:** 2019-07-23

**Authors:** Salim Gallouj, Niema Aqil, Taoufiq Harmouch, Fatima Zahra Mernissi

**Affiliations:** 1 Service de Dermatologie, CHU Hassan II de Fès, Maroc; 2 Service de Pathologie, CHU Hassan II de Fès, Maroc

**Keywords:** Carcinome basocellulaire, néoplasie, chirurgie micrographique de Mohs, prise en charge, récidive, Basal cell carcinoma, neoplasia, Mohs micrographic surgery, management, recidivism

## Abstract

La chirurgie micrographique de Mohs (CMM) est une technique permettant le contrôle histologique per-opératoire de la totalité de la marge d'exérèse chirurgicale des tumeurs malignes. Le but de ce travail est de valider l'intérêt de la CMM dans la guérison maximale des carcinomes basocellulaires (CBC) chez nos malades. Sur une période de 5 ans, nous avons retenu 29 patients présentant un CBC de la face. La médiane d'âge était de 45,8 ans (12-80). Le sex-ratio H/F était de 1,23. Une seule étape était nécessaire pour une exérèse complète dans 51% des cas. Le recours à 3 étapes était nécessaire dans 14% des cas. La durée moyenne de l'intervention a été d'une heure pour les cas ne nécessitant qu'une seule étape. Aucune complication n'a été rapportée en post opératoire et les suites étaient simples. Le résultat esthétique et fonctionnel était satisfaisant. Aucune récidive n'a été notée. Le CBC représente environ 80% de tous les cancers de la peau. La décision de traiter le CBC par CMM est basée sur trois variables: le siège et la taille de la tumeur, l'aspect histologique avec la définition de la marge d'exérèse et le caractère récidivant. La CMM est actuellement la méthode la plus efficace pour le traitement du CBC et permet la préservation du maximum de tissu sain. C'est une méthode chirurgicale sûre et reproductible, fondée sur un travail d'équipe et adaptée au traitement des CBC à haut risque de récidive. Les résultats esthétiques et fonctionnels sont satisfaisants. Le taux de récidive à 5 ans est 10 fois inférieur aux autres méthodes.

## Abstract (en)

Mohs micrographic surgery (MMS) is a technique allowing for intraoperative histologic assessment of surgical margin of malignant tumors. This study aims to highlight the role of MMS in the achievement of radical healing of basal cell carcinoma (BCC) in our patients. We conducted a study of 29 patients with basal cell carcinoma of the face over a period of 5 years. The median age of patients was 45.8 years (12-80). The sex-ratio M/F was 1.23. One-stage surgical procedure was sufficient to obtain complete resection in 51% of cases. Three-stage surgical procedure was needed in 14% of the cases. The mean duration of one-stage surgical procedure was 1 hour. No complications were reported in the postoperative period and sequelae were simple. The aesthetic and functional result was satisfactory. No recurrence was noted. BCC accounts for approximately 80% of all skin cancers. The decision to use MMS to treat BCC is based on three variables: the seat and the size of the tumor, its histological appearance with the identification of resection margin and its recurrent nature. MMS is currently the most effective method in the treatment of BCC and allows maximum healthy tissue preservation. It is a safe and repeatable surgical procedure based on team work and adapted to the treatment of patients with BCC who are at high risk of recidivism. The aesthetic and functional results are satisfactory. Recurrence rate at 5 years is 10 times lower than with the other methods.

## Introduction

Le carcinome basocellulaire (CBC) est le cancer cutané le plus fréquent, il constitue 70% à 80% de l'ensemble des tumeurs de la peau [1]. C'est une tumeur à potentiel localement invasif sans risque de métastases à distance mais à haut risque de récidive notamment en l'absence d'exérèse complète [2]. Le taux de CBC a été estimé à 47% de l'ensemble des tumeurs cutanées selon les données d'une étude réalisée au CHU Hassan II de Fès [3]. La chirurgie micrographique de Mohs (CMM) est la technique de choix dans la prise en charge de cette entité histopathologique [2,4]. Elle a l'avantage de permettre un résultat esthétique et fonctionnel très satisfaisant. Le taux de récidive tumorale à 5 ans après CMM est 10 fois inférieur à toutes les autres méthodes [2]. L'objectif de ce travail est de valider l'intérêt de la CMM dans la prise en charge des CBC cutanés, avec une évaluation de son efficacité dans la prévention de la récidive et la préservation optimale du tissu sain chez nos malades.

## Méthodes

On a mené une étude prospective, monocentrique et descriptive. Ce travail a été conduit sur une période de 5 ans entre août 2011 et septembre 2016 au service de dermatologie du CHU Hassan II de Fès au Maroc et a inclus tous les patients qui se sont présentés en consultation externe pour CBC localisé au niveau de la face. Le diagnostic de CBC a été orienté par la dermoscopie et/ou confirmé par l'histologie. Ont été inclus dans ce travail, les patients ayant des localisations à haut risque de récidive, les formes histologiques agressives et les formes mal limitées ou récidivantes. Les CBC de taille >3,5cm et les patients perdus de vue au suivi ou ayant refusé de participer ont été exclus de l'étude. Tous les patients ont bénéficié d'une dermoscopie avant la chirurgie. Pour chaque patient, un debulking a été réalisé pour l'obtention d'une galette avec identification et schématisation des prélèvements sur la carte Mohs. Chaque galette a été transportée sur une plaque de polystyrène au laboratoire d'anatomie pathologique du CHU Hassan II de Fès, des coupes fines ont été réalisées et colorées à l'Hematoxyline Eosine Safran (HES) puis visualisées au microscope. Si les berges apparaissent envahies, une exérèse limitée à la zone positive cartographiée sur le schéma est réalisée et analysée suivant les mêmes étapes. Cette manœuvre a été répétée jusqu'à obtention de berges complètement saines. Pour chaque malade inclus, l'ensemble des données épidémio-cliniques, dermoscopiques et histologiques ont été recueillies à la première consultation, pendant le geste chirurgical (type d'anesthésie, nombre d'étapes nécessaires, type de complications et type de reconstruction cutanée) et au cours du suivi ultérieur. Après chirurgie de Mohs, les patients ont été régulièrement suivis en consultation spécialisée à 3 mois, 6 mois puis une fois par an pendant 5 années consécutives. L'absence de récidive locale a été retenue après 5 ans de suivi.

## Résultats

Entre août 2011 et septembre 2016, 29 cas de CBC ont été colligés au service de dermatologie du CHU Hassan II de Fès.

**Age**: l'âge moyen des patients de notre étude était 45,8 ans (12-80).

**Sexe**: il n'a pas été noté une prédominance de sexe dans notre étude; avec un sex-ratio (H/F) de 1,23.

**Mode de découverte**: quatre formes récidivantes ont été rapportées dans notre série. Les 25 autres CBC inclus sont des CBC de novo.

**Topographie**: la localisation la plus fréquente était au niveau du nez (n=11; 38%). Quatre-vingt six pourcent (n=25) des CBC siégeaient en zone H.

**Histologie**: sur le plan histopathologique, les formes nodulaires et infiltrantes étaient les formes prédominantes dans notre étude avec respectivement des taux de 48% et 27%. Il n'a pas été rapporté de forme sclérodermiforme ni métatypique. L'infiltration péri-nerveuse n'a été retrouvée chez aucun patient.

**Technique chirurgicale**: une seule étape de la chirurgie de Mohs a été suffisante chez 51% des patients (n=15) avec un temps chirurgical d'une heure seulement. Le recours à une deuxième étape voire une troisième étape a été nécessaire chez respectivement 35% (n=10) et 14% (n=4) des malades. Le temps chirurgical est passé en moyenne à 2 heures et demi chez les patients ayant nécessité un recours à une deuxième étape et 3,1 heures chez les patients ayant bénéficié de 3 étapes. Le recours à plus de 3 étapes ou au slow Mohs n'a été indiqué chez aucun malade. Quatre types de reconstruction ont été proposés aux patients de notre série: dix huit (18) patients ont bénéficié de lambeaux d'avancement, de rotation ou de transposition. La cicatrisation dirigée a été préconisée dans 4 cas. Trois patients ont bénéficié d'une greffe de peau totale. La suture directe a été réalisée chez 4 cas.

**Figure 1:**
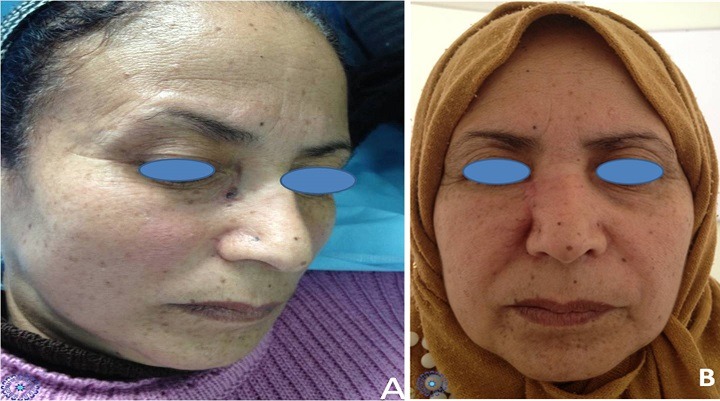
A) présentation clinique d'un carcinome basocellulaire évoluant depuis une année; B) résultat esthétique deux ans après un lambeau d'avancement palpébral en L

**Figure 2:**
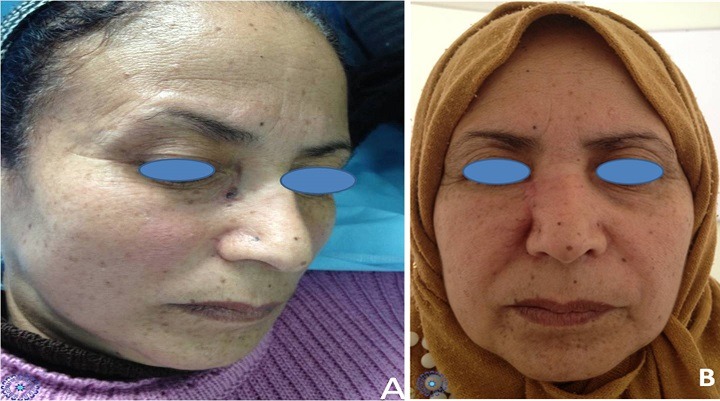
A) présentation clinique d'un carcinome basocellulaire évoluant depuis deux ans; B) résultat esthétique après une année

**Figure 3:**
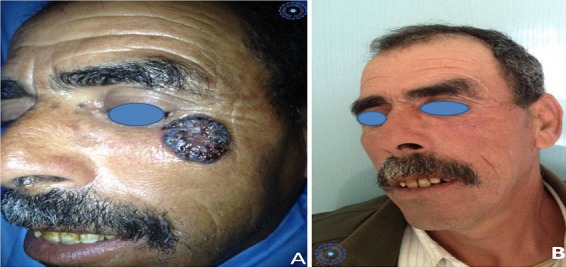
A) présentation clinique d'un carcinome basocellulaire évoluant depuis deux ans; B) résultat esthétique après trois ans

**Evolution**: les suites opératoires ont été simples: aucune complication n'a été rapportée et le résultat esthétique et fonctionnel a été satisfaisant. Les figures illustrent la présentation clinique au moment du diagnostic (Figure 1) et le résultat esthétique (Figure 2) obtenu chez certains patients de notre série après un suivi régulier (Figure 3).

## Discussion

Le carcinome basocellulaire est une tumeur cutanée fréquente à malignité locale. La chirurgie micrographique de Mohs est le traitement de choix de cette entité néoplasique, elle a permis de réduire considérablement le taux de récidive. L'objectif de ce travail est de présenter l'expérience du service de dermatologie du CHU Hassan II de Fès dans la prise en charge des CBC par la chirurgie micrographique de Mohs. Dans notre série, la médiane d'âge des patients était de 45,8 ans. La comparaison avec les données des séries de la littérature montre la survenue plus précoce du CBC dans notre population. En effet, l'âge moyen de découverte du CBC a été supérieur à 60 ans dans les autres études; il a été respectivement égal à 67,4; 72; 74 et 70,7 dans les séries de Van loo E *et al*. Wetzig *et al*. Alonso T *et al*. et Galimberti *et al.* [5-8]. Cela pourrait être d'une part expliqué par la particularité du climat au Maroc et le nombre de journées ensoleillées pouvant aller jusqu'à 300 jours/an mais aussi par l'absence de la culture de protection cutanée contre les UV justifiant l'augmentation des efforts en matière d'éducation et de sensibilisation quant à l'intérêt de l'usage des écrans solaires. Comme dans les autres séries de la littérature, il n'a pas été noté de prédominance de sexe parmi nos malades. Le sex-ratio a été égal à 1,2 dans notre série et respectivement égal à 1,01; 0,76 et 0,8 dans les études de Veronese *et al.* Angulo *et al.* et Wetzig *et al.* [6,9,10].

Sur le plan topographique, le nez était la localisation la plus fréquente dans notre série (38%). Cela rejoint les données de la littérature: le nez était la localisation la plus rapportée dans les autres séries avec des taux de 47,8%, 56,8% et 52,4% décrits respectivement dans les séries de J. Angulo *et al*. Blazaquez Sanchez et Alonso *et al*. Cela pourrait être expliqué par le fait que le nez représente la région la plus exposée aux UV de tout le visage [7,10,11]. La zone H concerne les régions périorificielles du visage (périoculaire, périauriculaire, nasale et périnasale, péribuccale, temporale). Il a été établit que c'est une zone à haut risque de survenue du CBC [2,12]. En effet, l'atteinte de la zone H était de l'ordre de 86% dans notre étude. Cela convient aux données décrites par Veronese *et al*. (83,8%), Smeets *et al*. (89%) et Alonso *et al.* (71,4%) [7,9,13]. Il reste à noter que ce taux a été moins important dans les études de Wetzig et coll. (54,25%), Leibovitch *et al.* (59,3%) et Blechman *et al.* (33,4%) [6,14,15]. Le type histologique le plus rapporté dans notre série était le sous type nodulaire (48%). Ceci rejoint les données rapportées dans les études de Alonso *et al*. et Lim *et al*. avec respectivement des taux de 52,4% et 40% [7,16]. Les formes agressives ont été diagnostiquées chez 44% de nos malades incluant les sous-types infiltrants (27%) et micronodulaires (17%). Aucun cas de CBC sclérodermiforme n'a été colligé dans notre étude. Le CBC a été plus agressif dans les séries d'Angulo *et al.* Veronese *et al*. et Smeets *et al*. ayant rapporté respectivement des taux de 60,9%, 63,1% et 56%. Ceci peut être expliqué par le phototype clair de la population Australienne et Européenne et la localisation géographique de l'Australie [9, 10, 13]. Le recours à une seule étape d'exérèse chirurgicale a été suffisant dans 51% des cas chez nos patients. Il a été proche des taux rapportés dans des séries regroupant un nombre plus important de CBC. Lawrence *et al*. rapporte un taux de 56% pour une série de 1090 CBC [17]. Un taux de 53,7% a été mentionné dans l'étude de Wetzig *et al*. ayant colligé 862 CBC [6]. Dans la série d'Alonso *et al*. le recours à une seule tranche périphérique a été satisfaisant dans 45,2% des cas [7]. Quand la technique chirurgicale se limite à une seule étape, cela permet d'optimiser encore plus la prise en charge du CBC en permettant une exérèse totale du tissu tumoral avec une préservation maximale du tissu sain. L'absence de délai pour une autre étape n'est pas incommodant pour le patient et lui épargne l'attente d'une autre étape. Le recours à une 2^ème^ et 3^ème^étape a été nécessaire respectivement chez 35% et 14% de nos patients et aucun malade n'a nécessité plus de 3 étapes. Dans tous les cas, le bénéfice de la chirurgie de Mohs reste bien établi quelque soit le nombre d'étapes indispensables pour l'exérèse tumorale complète [18-20]. La CMM permet une reconstruction esthétique meilleure, un bon résultat fonctionnel et un faible risque de récidive [10,16,17].

La durée minimale de suivi de nos patients était de 1an et demi, elle a dépassé 5 ans dans 24 cas. Il n'a pas été noté de récidive dans notre série. Cela pourrait être en rapport avec la qualité du geste chirurgical et l'absence d'envahissement périnerveux à l'étude histopathologique. En effet, l'invasion péri-nerveuse, bien que peu fréquente, a été retenue comme facteur de risque de survenue de récidive à 5 ans et comme signe d'agressivité de la tumeur [21]. Chez nos malades, les marges d'excision tumorale étaient saines. Les facteurs pronostiques majeurs de récidive sont la localisation en Zone H, la taille tumorale supérieure à 20mm quelque soit la localisation et les sous-types histologiques sclérodermiformes et micronodulaires [12]. Dans notre série, 86% des CBC étaient localisés dans la zone H, la taille tumorale était comprise entre 2 cm et 3,5 cm et le caractère infiltrant a été de l'ordre de 27%; il reste nécessaire de continuer une surveillance régulière chez nos malades pour une meilleure évaluation du taux de récidive à long terme [13,21-27]. Comparée à la chirurgie conventionnelle, la chirurgie micrographique de Mohs assure les taux les plus élevés de guérison des CBC tout en permettant une conservation maximale du tissu sain. Ce taux a été de l'ordre de 98% dans l'étude de Lawrence *et al*. [17]. La chirurgie conventionnelle se base sur des coupes verticales de la tumeur; les limites d'exérèse sont définies à l'aveugle en fonction du degré de malignité de la tumeur, elles sont comprises entre 3 et 4 mm pour la tumeur de faible risque et sont beaucoup plus larges en ce qui concerne les tumeurs à haut risque. C'est-à-dire que cette technique ne permet pas de précision en ce qui concerne l'envahissement des berges, son contrôle est estimé par un taux de 1 à 2% seulement ce qui peut retentir sur les taux de guérison pour cette technique. La chirurgie conventionnelle sacrifie le tissu sain pour assurer le résultat de guérison. Par contre, la technique de Mohs guide avec exactitude l'acte chirurgical; l'excision n'intéressera que le tissu tumoral et les marges d'exérèse ne dépasseront pas 2 mm autour de la perte de substance. Elle se base sur l'étude micrographique de coupes fines et cartographiées, elle permet donc un contrôle complet des marges d'exérèse. Ainsi, la conservation du tissu sain est maximale. Les taux de récidive pour cette technique sont nettement diminués à 5 ans, ils sont 10 fois inférieures par rapport à la chirurgie conventionnelle [28-31].

## Conclusion

Bien que l'incidence du CBC au Maroc reste mal connue, notre étude a montré que cette tumeur est assez fréquente dans notre pays. La chirurgie micrographique de Mohs a révolutionné la prise en charge de cette entité néoplasique. Fondée sur la collaboration entre le dermatologue et l'anatomopathologiste, elle a permis de réduire considérablement le taux de récidive à long terme. Dans notre étude, il n'a pas été rapporté de cas de récidive et les résultats esthétique et fonctionnel étaient satisfaisants. La surveillance continue de nos patients permettra d'évaluer l'efficacité à long terme de cette technique.

### État des connaissances actuelles sur le sujet

- Le carcinome basocellulaire est une tumeur cutanée fréquente à malignité locale;
- La chirurgie micrographique de Mohs est le traitement de choix;
- La chirurgie micrographique de Mohs a permis de réduire considérablement le taux de récidive.

### Contribution de notre étude à la connaissance

- C'est la première étude réalisée au Maroc;
- Cette étude a permis de valider l'intérêt de la chirurgie micrograhique de Mohs dans le traitement de carcinome basocellulaire.

## Conflits des intérêts

Les auteurs ne déclarent aucun conflit d'intérêts.
